# Dynamic chromatin accessibility and transcriptional landscapes of porcine kidney cells during pseudorabies virus infection

**DOI:** 10.3389/fimmu.2026.1773053

**Published:** 2026-02-04

**Authors:** Songbai Yang, Mingyang Dong, Haixin Shi, Xiangchen Li, Han Wang, Xiaolong Zhou, Ayong Zhao

**Affiliations:** Key Laboratory of Applied Technology on Green-Eco-Healthy Animal Husbandry of Zhejiang Province, College of Animal Science and Technology, College of Veterinary Medicine, Zhejiang A&F University, Hangzhou, China

**Keywords:** accessible chromatin, ATAC-seq, pseudorabies virus, RNA-seq, transcription factors

## Abstract

**Background:**

Pseudorabies virus (PRV) is a major swine pathogen that causes substantial economic losses. The dynamic remodeling of host cell chromatin plays a pivotal role during viral infections. However, the epigenetic mechanisms underlying PRV-host interactions remain unclear.

**Methods:**

This study integrates ATAC-seq and RNA-seq to investigate the dynamic changes in host chromatin accessibility and gene transcription during PRV infection. The accessible chromatin regions were analyzed for enrichment in genomic features and transcription factor binding motifs. RNA-seq data were used to identify differentially expressed genes and dysregulated pathways. The two datasets were integrated to examine correlations between chromatin accessibility and gene expression.

**Results:**

PRV infection induces a genome-wide elevation in host chromatin accessibility, which progressively intensifies throughout the course of infection. These accessible chromatin regions are predominantly enriched in promoters and binding motifs for bZIP family transcription factors, such as BATF, ATF3, and AP-1, suggesting these transcription factors may play an important role in PRV infection. RNA-seq analysis reveals that PRV infection significantly dysregulates genes involved in metabolic and immune response pathways, with extensive transcriptional suppression observed in the late stages. Integration of ATAC-seq and RNA-seq data demonstrates that chromatin accessibility is positively correlated with gene expression for the majority of differentially expressed genes. However, certain genes exhibit discordant regulation, implying the existence of more complex regulatory mechanisms.

**Conclusion:**

This study provides valuable epigenetic insights into the PRV-host interaction and establishes a theoretical framework for developing novel antiviral strategies.

## Introduction

Pseudorabies virus (PRV), also known as Suid herpesvirus 1, belongs to the genus *Varicellovirus* of the family *Herpesviridae*. The PRV genome is approximately 143 kb in length and encodes more than 70 proteins that participate in viral infection, immune evasion, and neurotropism (1). PRV can infect many species, such as pigs, cattle, sheep, dogs, cats, and rodents (2). Pigs are the natural host and main reservoir for the virus. In pigs, PRV infection can lead to reproductive failure in sows, severe encephalitis, and a mortality rate of up to 100% in newborn piglets (3). There have been a number of occurrences of human PRV infection in the last few years, which suggests that PRV could possibly be a hazard to public health (4). Even while many affluent countries have been able to get rid of PRV by vaccination, surveillance, and culling programs, the virus is still common in some parts of the world (5). Since 2011, PRV variant strains in China have inflicted significant economic losses on the swine industry (6). Consequently, unraveling the pathogenic processes of PRV is essential for the development of novel therapeutic and preventive treatments.

Gene expression is tightly regulated by the degree of chromatin packaging. In eukaryotes, genomic DNA associates with histone proteins to form chromatin. The fundamental structural unit of chromatin is the nucleosome, which consists of DNA wrapped around histone octamers. Nucleosomes are further compacted into higher order condensed fiber structures with the aid of linker histone H1 (7, 8). Chromatin accessibility is how easy it is for big molecules like transcription factors to get to DNA inside chromatin (9). Chromatin accessibility is quite dynamic; it can go from a closed, condensed state to an open, nucleosome-depleted state that permits transcription factors to bind, or the other way around (8). The relationship between transcription factors and DNA is directly related to how accessible chromatin is, since most transcription factors prefer to bind to open chromatin regions. Transcription factors dynamically compete with histones and recruit ATP-dependent chromatin remodeling complexes to modulate nucleosome occupancy and local DNA accessibility (7). Chromatin accessibility is essential for the regulation of gene expression, with open sections largely representing cis-regulatory elements such as enhancers and promoters, where transcription factors bind to modulate gene expression (10). So, chromatin accessibility shows how well transcription factors can bind to genes in a certain area and how well genes can control other genes (8). This concept forms the basis for investigating differences in transcription factor binding and alterations in cell state by profiling genome-wide chromatin accessibility.

ATAC-seq (Assay for Transposase-Accessible Chromatin with high-throughput sequencing) is a robust method for delineating genome-wide chromatin accessibility (11). This method uses the Tn5 transposase to cleave open chromatin regions and add sequencing adapters at the same time. Then, high-throughput sequencing and analysis are used to find open chromatin regions, transcription factor binding sites, and nucleosome placement (12). ATAC-seq offers major advantages in terms of speed and simplicity as the procedure can generally be completed within a few hours. Furthermore, it demonstrates high sensitivity superior to DNase-seq and FAIRE-seq methods by requiring only a minimal number of starting cells ranging from 500 to 50,000 (11, 13, 14). Given its broad applicability, ATAC-seq is widely employed to characterize cell type specific regulatory elements, including promoters and enhancers. Furthermore, it plays a pivotal role in elucidating the epigenetic mechanisms involved in cell differentiation, development, and disease pathogenesis (13, 15–17).

Viral infection can modulate host chromatin accessibility and thereby alter gene expression (18). These alterations can occur either locally or globally. For example, herpes simplex virus type 1 (HSV-1) increases chromatin accessibility downstream of genes by interfering with transcription termination (DoTT), which is closely linked to the production of downstream transcripts (19). In contrast, infection with modified vaccinia virus Ankara (MVA) results in a worldwide reduction in chromatin accessibility and condensation of host chromatin, which is associated with the downregulation of immune-related genes (20). Additionally, Epstein-Barr virus (EBV) can use noncoding RNAs to target chromatin areas with low accessibility, inhibiting RNAPII-associated chromatin loops and thereby downregulating genes implicated in cell cycle progression and immunological responses (21). Integrative analysis of ATAC-seq and RNA-seq data facilitates systematic correlation between differentially accessible chromatin regions and differentially expressed genes (DEGs), aiding in the identification and prediction of transcription factors and their binding sites implicated in virus-induced host gene regulation.

Although viral infection has been shown to profoundly reshape host chromatin accessibility, it’s still not understood how PRV changes chromatin accessibility in host cells or how this affects gene expression. In this study, PK15 cells infected with PRV were sampled at 0, 4, 8, and 12 h post-infection for combined ATAC-seq and RNA-seq analyses. This study aims to comprehensively characterize the dynamics of chromatin accessibility and gene expression in host cells during PRV infection, thereby providing new insights into the epigenetic regulatory mechanisms underlying PRV-host interactions.

## Materials and methods

### Cell culture and virus infection

PK15 cells were obtained from the China Center for Type Culture Collection (Cat^#^ GDC0061, CCTCC). Cells were cultured in Minimum Essential Medium (MEM, Cat^#^ BC-M-020, BioChannel) supplemented with 10% fetal bovine serum (FBS, Cat^#^ A5256501, Gibco) and 1% non-essential amino acids (Cat^#^ 11140050, Gibco). All cells were maintained at 37 °C with 5% CO_2_.

PK15 cells were seeded in 6-well plates, and when cells reached approximately 90% confluence, they were infected with PRV. Prior to infection, cells were washed twice with PBS (Cat^#^ BC-BPBS-01, BioChannel). The cells were then infected with PRV (MOI = 0.1) and incubated for 1 h to allow viral adsorption. After adsorption, the cells were washed three times with PBS and maintained in medium containing 2% FBS. The infection time was calculated from when the virus was initially added to the cells. Samples were collected at 4 h, 8 h, and 12 h post-infection, and an uninfected control group (0 h) was included. Three biological replicates were performed for each time point. The PRV strain used in this study was the ZJ (Zhejiang) strain (22).

### Immunofluorescence

Cells that had been infected with PRV at different times were washed twice with PBS and then fixed in 4% paraformaldehyde (Cat^#^ P1110, Solarbio) for 15 minutes at room temperature. Cells were washed twice with PBS for 5 minutes each time, and then they were permeabilized with 0.2% Triton X-100 (Cat^#^ HY-Y1883A, MedChemExpress) for 15 minutes at room temperature. After that, cells were washed twice with PBS. To block nonspecific binding, cells were incubated with 5% BSA (Cat^#^ 0332-100G, VWR) in PBS for 1 h at room temperature. After blocking, cells were incubated with anti-PRV rabbit polyclonal antibody (1:50, Cat^#^ PA1-081, Thermo Scientific) overnight at 4 °C. The primary antibody was discarded, and cells were washed with PBS three times for 10 minutes each time. Then, Alexa Fluor 488-conjugated goat anti-rabbit secondary antibody (1:500, Cat^#^ A-11008, Thermo Scientific) was used for 1 h at room temperature in the dark. After getting rid of the secondary antibody, the cells were washed three times with PBS in the dark. Finally, DAPI staining solution (Cat^#^ AC0131S, AccuRef Scientific) was added, and cells were incubated for 10 minutes at room temperature in the dark. After staining, cells were washed three times with PBS, each time for five minutes. Fluorescent images were acquired using a Nikon Ts2R fluorescence microscope (Nikon).

### ATAC-seq library construction

At different time points after PRV infection, cells were washed twice with PBS and then digested with trypsin-EDTA (Cat^#^ BC-CE-005, BioChannel). Cells were transferred to sterile centrifuge tubes, centrifuged at 800 rpm for 5 minutes, and the supernatant was discarded. After washing with PBS, cells were centrifuged again and the supernatant was discarded. The cells were resuspended in PBS, and 50,000 cells per sample were used for nuclear extraction. Briefly, cells were suspended in a cell lysis buffer containing 50 mM HEPES (Cat^#^ 15630080, Gibco), 140 mM NaCl (Cat^#^ AM9759, Invitrogen), 1 mM EDTA (Cat^#^ AM9260G, Invitrogen), 10% glycerol (Cat^#^ G7757-1L, Sigma-Aldrich), 0.5% NP-40 (Cat^#^ 85124, Thermo Scientific), and 0.25% Triton X-100 (Cat^#^ 28314, Thermo Scientific), with added protease inhibitors (Cat^#^ 4693159001, Roche). The extracted nuclei were incubated in 50 μL transposase reaction mixture (ATAC-seq library prep kit, Yingzi Gene) at 37 °C for 1 h. The transposed product was purified using a DNA purification and concentration kit (Cat^#^ TD413-50, Jianshi Biotechnology). PCR amplification was performed using NEBNext High-Fidelity 2X PCR Master Mix (Cat^#^ M0541L, NEB) and adapter primers. The amplification program was as follows: 72 °C for 3 m, 98 °C for 30 s, followed by 9 cycles of 98 °C for 15 s, 60 °C for 30 s, and 72 °C for 30 s, and a final extension at 72 °C for 5 m. The amplified products were purified using KAPA Pure Beads (Cat^#^ KS8002, KAPA Biosystems), and fragment selection was performed. The library fragment size and concentration were analyzed using an Agilent 2100 Bioanalyzer (Agilent Technologies), and sequencing was conducted using the Illumina NovaSeq 6000 platform (Illumina) with PE150 sequencing.

### RNA-seq library construction

At different time points after PRV infection, total RNA was extracted from cells using the FastPure Cell/Tissue Total RNA Isolation Kit V2 (Cat^#^ RC112-00, Vazyme) according to the manufacturer’s instructions. RNA-seq libraries were prepared using the VAHTS Universal V8 RNA-seq Library Prep Kit for MGI (Cat^#^ NRM605-02, Vazyme). First, 500 ng of total RNA was enriched for mRNA using VAHTS mRNA Capture Beads (Cat^#^ N401-02, Vazyme), and the RNA was fragmented to a size of 300–400 bp. Then, the RNA was reverse transcribed into cDNA, and sequencing adapters were added to the ends of the cDNA. PCR amplification was performed using amplification primers from the adapters. Amplified PCR products were purified using KAPA Pure Beads, and fragment selection was performed. The library fragment size and concentration were analyzed using an Agilent 2100 Bioanalyzer, and sequencing was performed on the Illumina NovaSeq 6000 platform with PE150 sequencing.

### ATAC-seq data analysis

The raw sequencing data were quality controlled using fastp (version 0.23.2) (23), which removed adapter sequences, low-quality bases, unknown bases, and reads shorter than 5 bp after quality control. High-quality reads were aligned to the pig reference genome (Sscrofa11.1) using Bowtie2 (version 2.3.4.1) (24) with default parameters. To improve the accuracy of read alignment, since PK15 cells are PCV1-positive, a combined reference genome was constructed by appending the genomic sequences of PRV (KX423960.1) and Porcine circovirus 1 (PCV1, NC_001792.2) to the *Sus scrofa* host genome prior to alignment. Insert fragment length distributions were analyzed using samtools (version 1.12) (25), and sequencing reads around transcription start sites were analyzed using deepTools (version 3.5.1) (26) to assess library quality. Open chromatin regions were identified for each sample using MACS2 (version 2.1.1) (27). The distribution of peaks across chromosomes was visualized using Circos (version 0.69.9) (28). Genomic annotations of open chromatin regions were performed using the R package ChIPseeker (version 1.32.1) (29), prioritizing annotation categories in the following order: Promoter > 5’ UTR > 3’ UTR > Exon > Intron > Downstream > Distal Intergenic. Chromatin accessibility signal intensity for open chromatin regions was quantified using Homer (v4.11.1) (30) with the makeTagDirectory and annotatePeaks.pl commands. Transcription factor binding motif analysis was also performed using the Homer findMotifsGenome.pl tool to predict genomic motifs and enrich known annotated motifs. Differential peak identification was conducted using DESeq2 (version 1.36.0) (31), with significant chromatin accessibility differences determined by |log2FC| > 1 and adjusted P value < 0.05. Transcription factor footprint analysis was performed using RGT (version 0.12.3) (32). Briefly, footprint regions were identified using the rgt-hint footprinting command. Identified footprints were matched to known transcription factor motif databases using rgt-motif analysis matching. Finally, differential footprint analysis was conducted using rgt-hint differential to calculate activity scores for transcription factors under different conditions and generate footprint profiles.

### RNA-seq data analysis

Raw RNA-seq data were quality controlled using fastp (version 0.23.2), including removal of adapter sequences, low-quality reads, and sequences with a high proportion of unknown bases (N). Quality assessment was performed using fastQC (v0.11.9) (33). The pig reference genome (Sscrofa11.1) and corresponding annotation files were downloaded from the Ensembl database (https://asia.ensembl.org/Sus_scrofa/Info/Index). To construct a combined reference genome, the genomic sequences of PRV (KX423960.1) and PCV1 (NC_001792.2) were appended to the pig genome. Sequencing data were aligned to the reference genome using HISAT2 (version 2.2.1) (34), and transcript assembly was performed using Stringtie (version 2.2.1) (35). Gene expression levels were quantified using featureCounts (version 2.0.3) (36), with TPM (transcripts per kilobase million) as the expression metric. Differential expression analysis was performed using the R package DESeq2 (v1.36.0), with screening criteria of |log2FC| ≥ 1 and P value < 0.05. A 3D PCA scatter plot was generated using the R package scatterplot3d (version 0.3-44) (37).

### Gene enrichment analysis

Gene functional enrichment analysis of genes associated with differentially identified peaks from ATAC-seq and DEGs from RNA-seq was performed using the eggNOG database (version 5.0.2) (38) for homologous annotations. Gene Ontology (GO) and Kyoto Encyclopedia of Genes and Genomes (KEGG) pathway enrichment analyses were conducted using the R package clusterProfiler (version 4.4.4) (39).

### Integrated ATAC-seq and RNA-seq analysis

Differentially accessible chromatin regions identified from ATAC-seq were integrated with DEGs identified from RNA-seq data. In particular, detailed analysis was conducted on the data from 12 h post-infection (P12) compared to the uninfected control group (P0). Genes that overlapped between the differentially accessible chromatin regions and DEGs were selected to identify those with significant changes in both chromatin accessibility and gene expression. A quadrant plot was constructed using the R package ggplot2 (version 4.0.0.0) (40) to visualize the expression patterns of these genes. Signal tracks were visualized using pyGenomeTracks (version 3.9) (41) to show ATAC-seq signal, peak regions, gene annotations, and RNA-seq expression levels.

### RT-qPCR validation

cDNA was synthesized using the 5X All-In-One RT MasterMix kit (Cat^#^ G592, Applied Biological Materials). Specific primers (**Table 1**) and SYBR Green qPCR Master Mix (Cat^#^ HY-K0501, MedChemExpress) were used to perform quantitative PCR on the QuantStudio 3 real-time PCR system (Thermo Fisher Scientific). The reaction volume was 10 μL, containing 5 μL 2× SYBR Green Master Mix, 0.3 μL of each upstream and downstream primer (10 μM), 1 μL of cDNA template, and 3.4 μL of sterilized water. The amplification program consisted of an initial denaturation at 95 °C for 5 min, followed by 40 cycles of 95 °C for 15 s, 60 °C for 30 s, and 72 °C for 30 s. Each sample was analyzed in triplicate, with GAPDH used as the internal control gene. Relative gene expression was calculated using the 2^^-ΔΔCt^ method (42).

**Table 1:** RT-qPCR primer sequences.

Names	Primer sequence (5′→3′)	Size (bp)
FOSL1	F: AACCGAAGGAAAGAACTGACCG	254
	R: GAAAGGGAGATACAAGGCATAGGG	
RGS4	F: CCGTCCAGCAAACCAAAGAG	154
	R: GATTTGAGGAAGCGGCGATA	
LDLR	F: TCGGACATACATTTGATGGCAGAA	111
	R: TTGGAGGGCGGTCCTTTCAC	
ID3	F: CGTCTCCGGGAACTGGTAC	208
	R: GGCAGAAGGTCGTTTGGTC	
GAPDH	F: GGACTCATGACCACGGTCCAT	220
	R: TCAGATCCACAACCGACACGT	

## Results

### Effects of PRV infection on cellular morphology

**Figure 1:**
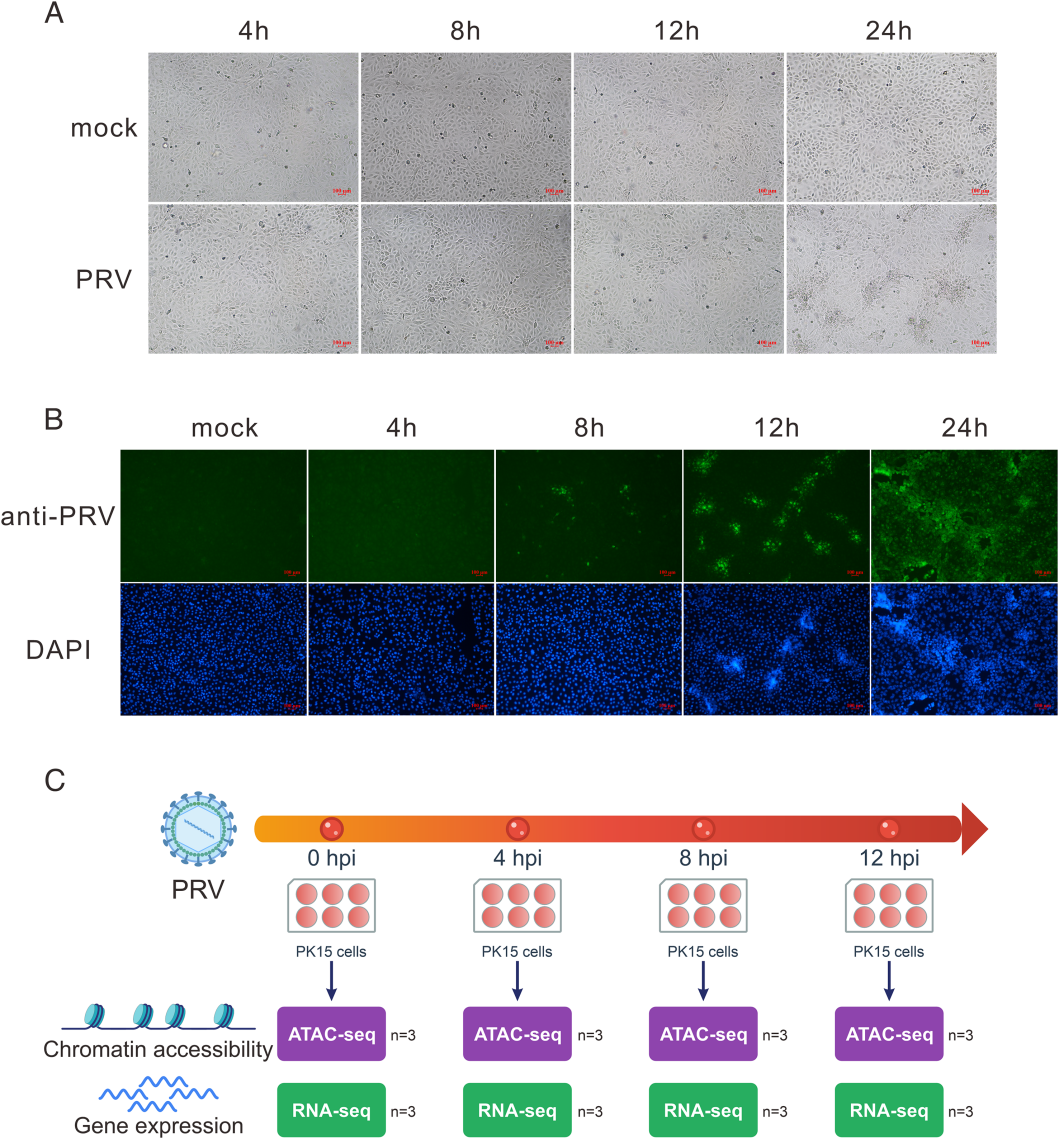
Morphological changes, viral infection dynamics, and experimental design of PRV-infected PK15 cells. **(A)** Morphological observation of PK15 cells at different time points post PRV infection and in the mock control group. Scale bar = 100 μm. **(B)** Immunofluorescence detection of PRV antigens (green) at indicated time points using an anti-PRV antibody, with nuclei stained by DAPI (blue). Scale bar = 100 μm. **(C)** Schematic overview of the experimental design. PK15 cells were collected at 0 h, 4 h, 8 h, and 12 h post PRV infection for ATAC-seq and RNA-seq analyses (n = 3).

We evaluated the effects of PRV infection on PK15 cell morphology and the progression of cytopathic effects (CPE) to determine the optimal sampling time points for multi-omics analysis. Accordingly, cells were harvested at 4, 8, 12, and 24 h post-infection (hpi), with uninfected cells serving as controls. The findings indicated that over the 4, 8, and 12 hpi intervals, the overall cell morphology was preserved, exhibiting no discernible indications of cell shrinkage or detachment. At 24 hpi, however, the monolayer’s integrity was disturbed, and typical symptoms of cellular damage, such as rounding, shrinkage, and aggregation, were seen (**Figure 1A**).

Immunofluorescence staining against viral proteins was performed to monitor viral replication. At 4 hpi, no fluorescence signal was detected, indicating that the virus had not yet accumulated significantly within the cells. By 8 hpi, distinct fluorescent signals appeared, indicating the commencement of active viral replication, but nuclear morphology remained largely unaltered. At 12 hpi, fluorescence intensity increased significantly, and the infected cells exhibited nuclear morphological abnormalities, characterized by irregular nuclear margins and chromatin condensation. At 24 hpi, nearly all cells exhibited strong fluorescence signals, indicating extensive viral spread and release via cell lysis. At this stage, nuclei appeared transparent and were accompanied by widespread, high-degree chromatin condensation (**Figure 1B**).

Given that PRV infection induces significant alterations in chromatin structure during the late stages, we selected time points (4, 8, and 12 hpi) where cells had not yet undergone obvious cytopathic effects or extensive chromatin condensation to perform ATAC-seq and RNA-seq analysis, using uninfected cells (0 h) as a control. This strategy aimed to capture the authentic genome-wide dynamic changes in chromatin accessibility during viral infection. Three biological replicates were included for each time point (**Figure 1C**).

### Peak identification and genomic distribution of ATAC-seq data

**Figure 2:**
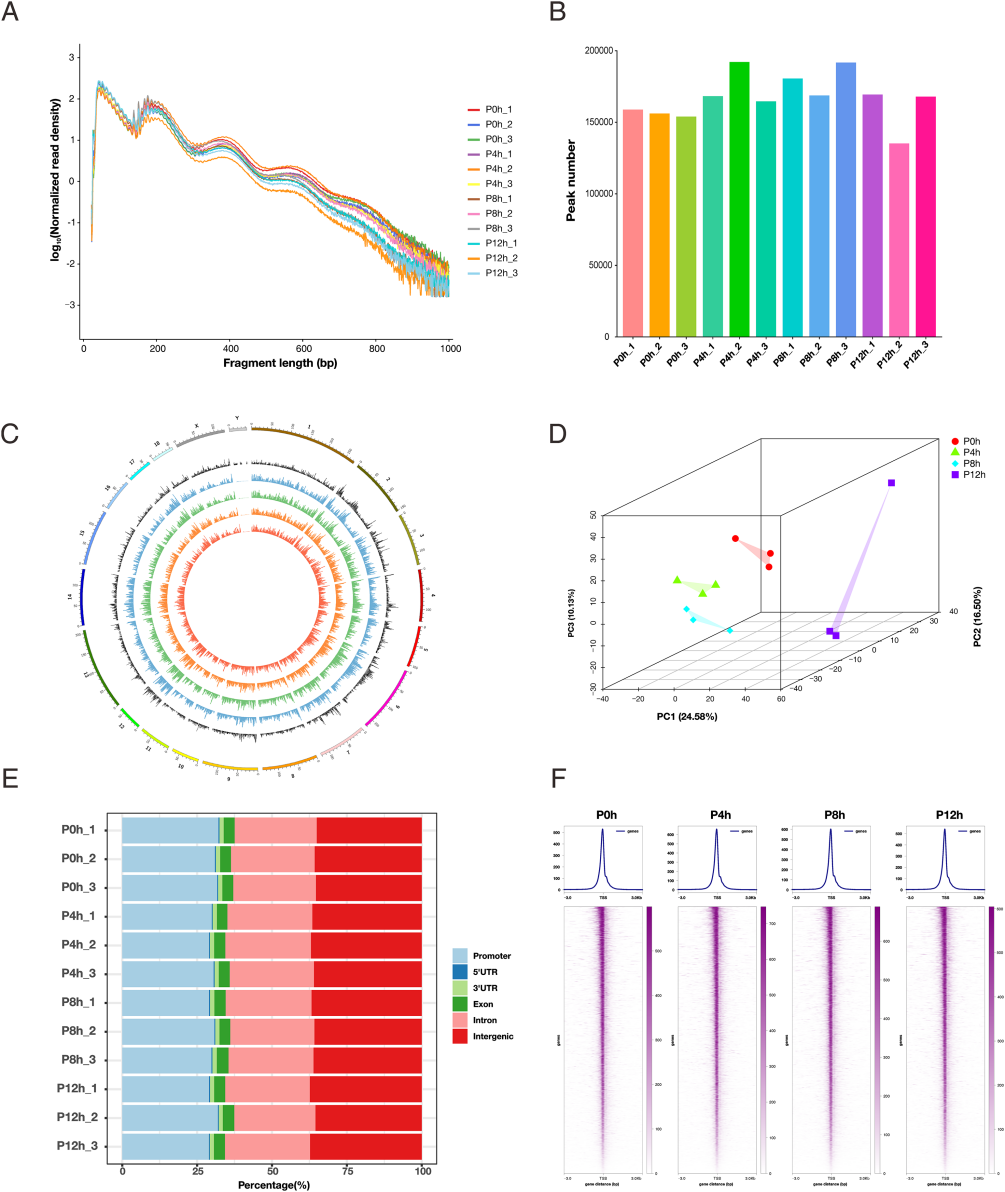
Quality assessment and genomic distribution of ATAC-seq data. **(A)** Fragment length distribution of ATAC-seq reads across all samples. **(B)** Number of accessible chromatin peaks identified in each sample. **(C)** Circos plot showing chromosomal distribution of peaks across samples. From the inner to the outer rings: peaks from sample P0, P4, P8, and P12; gene density; and chromosomes. **(D)** Principal component analysis (PCA) of ATAC-seq data illustrating the clustering of samples from different time points post PRV infection. **(E)** Genomic annotation of significant peaks showing their distribution in promoters, UTRs, exons, introns, and intergenic regions. **(F)** Transcription start site (TSS) enrichment analysis of ATAC-seq signals for each time point.

To characterize the dynamic changes in host chromatin accessibility during PRV infection, we performed ATAC-seq on PK15 cells at 0, 4, 8, and 12 hpi. Analysis of fragment length distribution revealed a typical nucleosome periodicity in all samples, with distinct peaks corresponding to nucleosome-free regions (NFRs, <100 bp), mononucleosomes (~200 bp), and dinucleosomes (~400 bp) (**Figure 2A**). This pattern indicates efficient chromatin fragmentation and high data quality. Subsequent peak calling for each time point showed that, compared to uninfected cells, PRV infection resulted in the detection of more accessible peaks at all post-infection time points, suggesting a global increase in host chromatin openness induced by the virus (**Figure 2B**).

Examination of the genomic distribution of peaks revealed that accessible regions were broadly distributed across the genome (**Figure 2C**). Principal Component Analysis (PCA) showed that samples clustered according to infection time points, indicating data consistency, with distinct separation observed between different stages (**Figure 2D**, **Supplementary Figure 2A**). Genomic annotation of significant peaks showed that the majority of open regions were located in promoters, introns, and intergenic regions, consistent with typical characteristics of mammalian ATAC-seq data (**Figure 2E**). Furthermore, transcription start site (TSS) enrichment analysis displayed strong and focused signal enrichment at TSSs for all samples (**Figure 2F**). This further confirms the reliability of the sequencing libraries and suggests that PRV infection may influence host gene transcription by regulating promoter regions and adjacent chromatin structures.

### Chromatin accessibility changes and motif analysis

**Figure 3:**
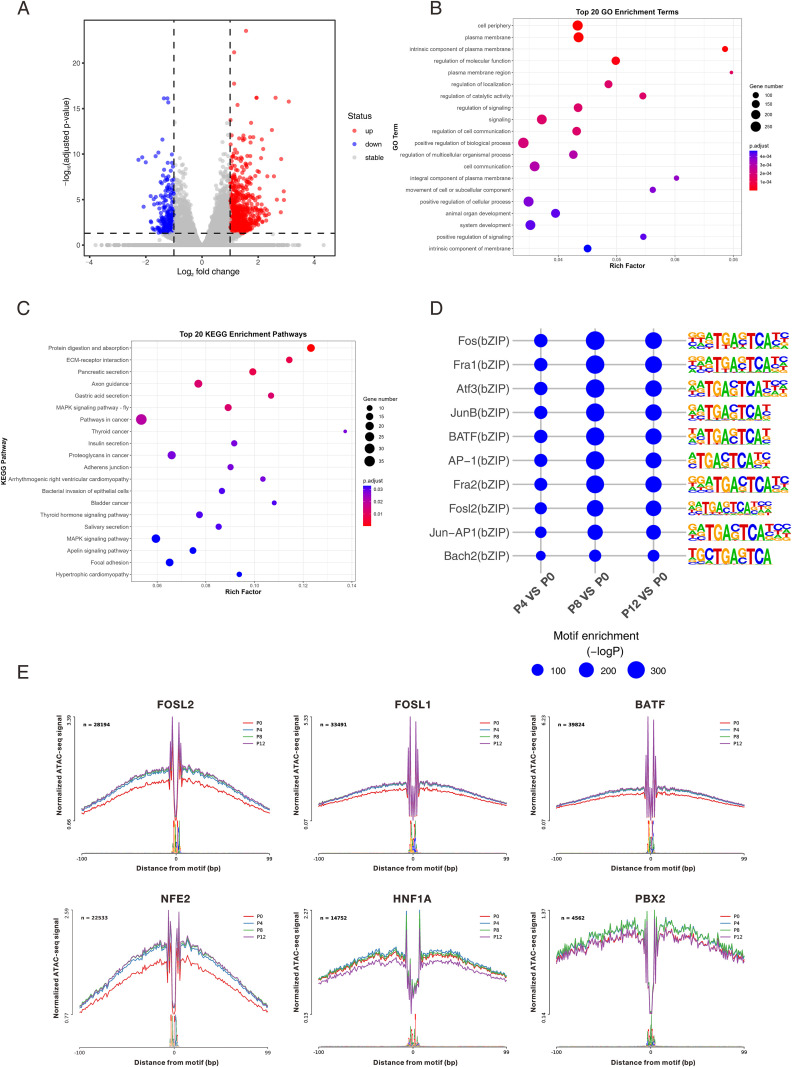
Chromatin accessibility and transcription factor motif analysis. **(A)** Volcano plot of differentially accessible chromatin peaks between PRV-infected (P12) and uninfected (P0) PK15 cells. **(B)** Gene Ontology (GO) enrichment analysis of DAR-associated genes at 12 h post-infection. **(C)** Kyoto Encyclopedia of Genes and Genomes (KEGG) pathway enrichment analysis of DAR-associated genes at 12 h post-infection. **(D)** Motif enrichment analysis of differentially accessible regions showing significantly enriched transcription factor binding motifs across different time points. **(E)** Footprint profiles of representative transcription factors (FOSL2, FOSL1, BATF, NFE2, HNF1A, and PBX2) in PRV-infected PK15 cells at 0 h, 4 h, 8 h, and 12 h post-infection.

We further identified differentially accessible regions (DARs) and performed transcription factor motif enrichment analysis. The results showed that PRV infection induced significant changes in numerous open chromatin regions at various time points. Notably, regions with increased accessibility were significantly more abundant than those with decreased accessibility, suggesting a progressive expansion of host chromatin accessibility as the infection proceeds (**Figure 3A**; **Supplementary Figures 1A, B**). To elucidate the potential biological functions of genes associated with these DARs, we performed GO enrichment analysis. At 4 hpi, DAR-associated genes were primarily involved in processes such as tube morphogenesis. By 8 hpi, enrichment was significant in extracellular matrix organization and cell junctions. By 12 hpi, the functional involvement of differentially accessible regions shifted toward broader cellular regulatory processes, including plasma membrane activities, regulation of localization, regulation of signaling, and regulation of cell communication (**Figure 3B**; **Supplementary Figures 1C, D**). Further KEGG pathway analysis revealed that DAR-associated genes were significantly enriched in several key cellular signaling pathways, including Focal adhesion, ECM-receptor interaction, and the MAPK signaling pathway (**Figure 3C**; **Supplementary Figures 1E, F**). These pathways are closely related to cell structural remodeling, sensing of external stimuli, and downstream signal transduction.

To investigate the key regulators driving these changes in chromatin accessibility, we performed motif enrichment analysis on the differential regions. The results indicated significant enrichment of binding motifs for bZIP family transcription factors at multiple time points, including Fos, Fra1, Atf3, JunB, BATF, AP-1, Fosl2, and Bach2 (**Figure 3D**). To verify whether the transcription factors corresponding to these enriched motifs exhibited changes in binding activity during PRV infection, we subsequently conducted transcription factor footprint analysis. The results demonstrated that the accessibility of binding sites for TFs such as FOSL1, FOSL2, BATF, and NFE2 continuously increased post-infection. Conversely, the footprint signal for HNF1A decreased markedly at 12 hpi, while PBX2 accessibility exhibited a pattern of initial increase followed by a decrease (**Figure 3E**). These findings suggest that PRV infection induces dynamic changes in the binding activity of these key transcription factors, which may act as upstream regulators directly participating in host chromatin opening and transcriptional reprogramming.

### Global transcriptomic changes induced by PRV infection

**Figure 4:**
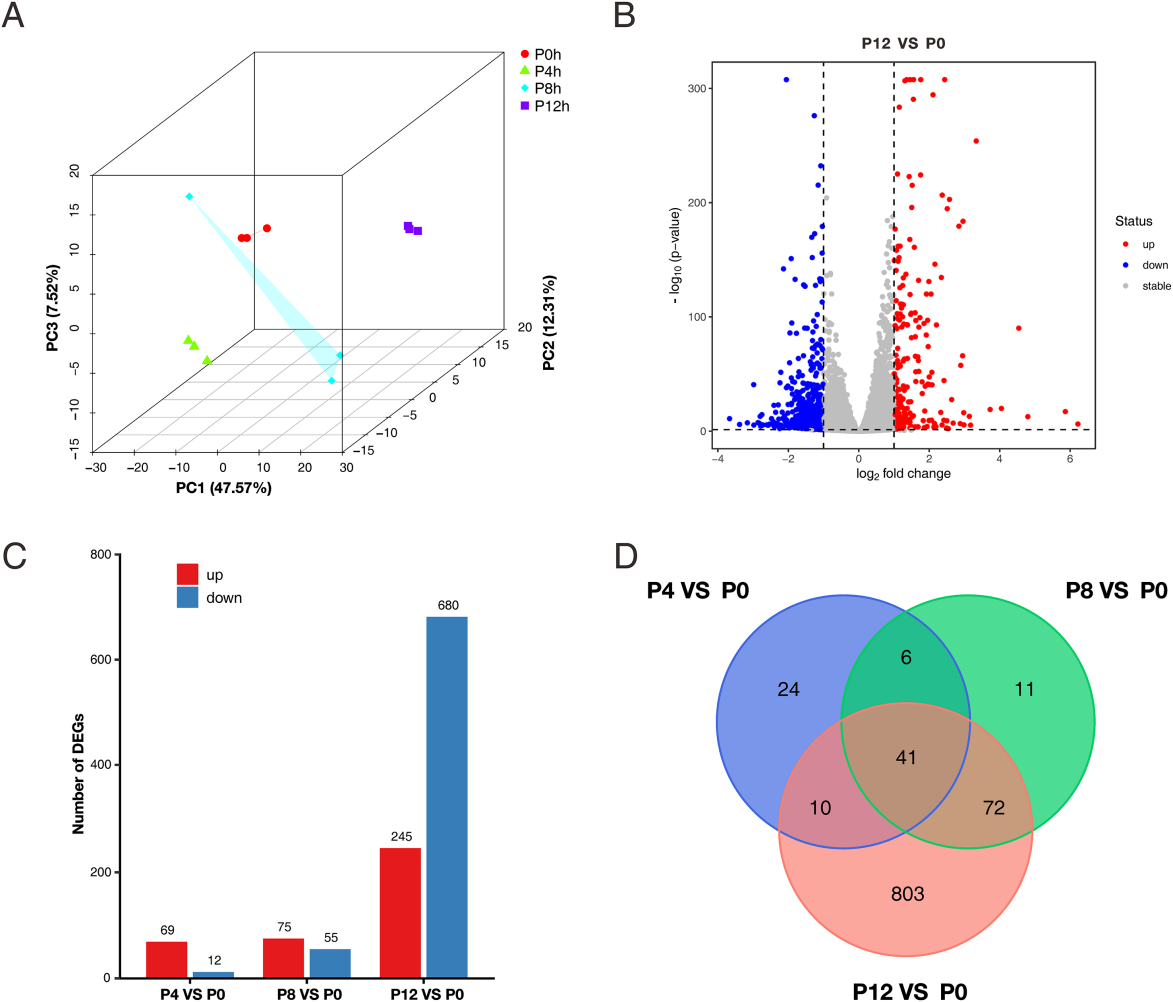
Transcriptomic analysis of PK15 cells at different time points post PRV infection. **(A)** Principal component analysis (PCA) of RNA-seq data showing the overall transcriptomic variation among PK15 cells at 0 h, 4 h, 8 h, and 12 h post PRV infection. **(B)** Volcano plot showing differentially expressed genes (DEGs) between PRV-infected (P12) and uninfected (P0) PK15 cells. Red and blue dots represent up- and down-regulated genes, respectively. **(C)** Number of up- and down-regulated DEGs identified at 4 h, 8 h, and 12 h post-infection compared with the mock control (P0). **(D)** Venn diagram showing the overlap of DEGs among different infection time points (P4 vs P0, P8 vs P0, and P12 vs P0).

Changes in chromatin accessibility typically regulate gene transcription by modulating transcription factor binding. To investigate whether the chromatin accessibility changes induced by PRV infection were accompanied by alterations in host gene transcription levels, we performed RNA-seq analysis on PK15 cells at the same infection time points (0, 4, 8, 12 hpi). The PCA results showed that the three biological replicates were well-clustered at each time point. One sample at 8 hpi was a little different from the other two in the same group, but the correlation coefficients were all greater than 0.99 (**Figure 4A**; **Supplementary Figure 2B**). Differential expression analysis revealed that the number of DEGs gradually increased with the duration of infection. At 4 and 8 hpi, respectively, 81 and 130 DEGs were found, with the majority of them being elevated genes. However, by 12 hpi, the number of DEGs increased significantly to 925. At this stage, transcriptional suppression became more pronounced, with the number of downregulated genes significantly exceeding that of upregulated genes (**Figures 4B, C**; **Supplementary Figure 3**). This shows that the host cell’s transcriptional response to viral infection is strongest at 12 hpi. The Venn diagram analysis revealed both unique and common DEGs at various time points, with 41 genes demonstrating differential expression at all three infection time points (**Figure 4D**).

**Figure 5:**
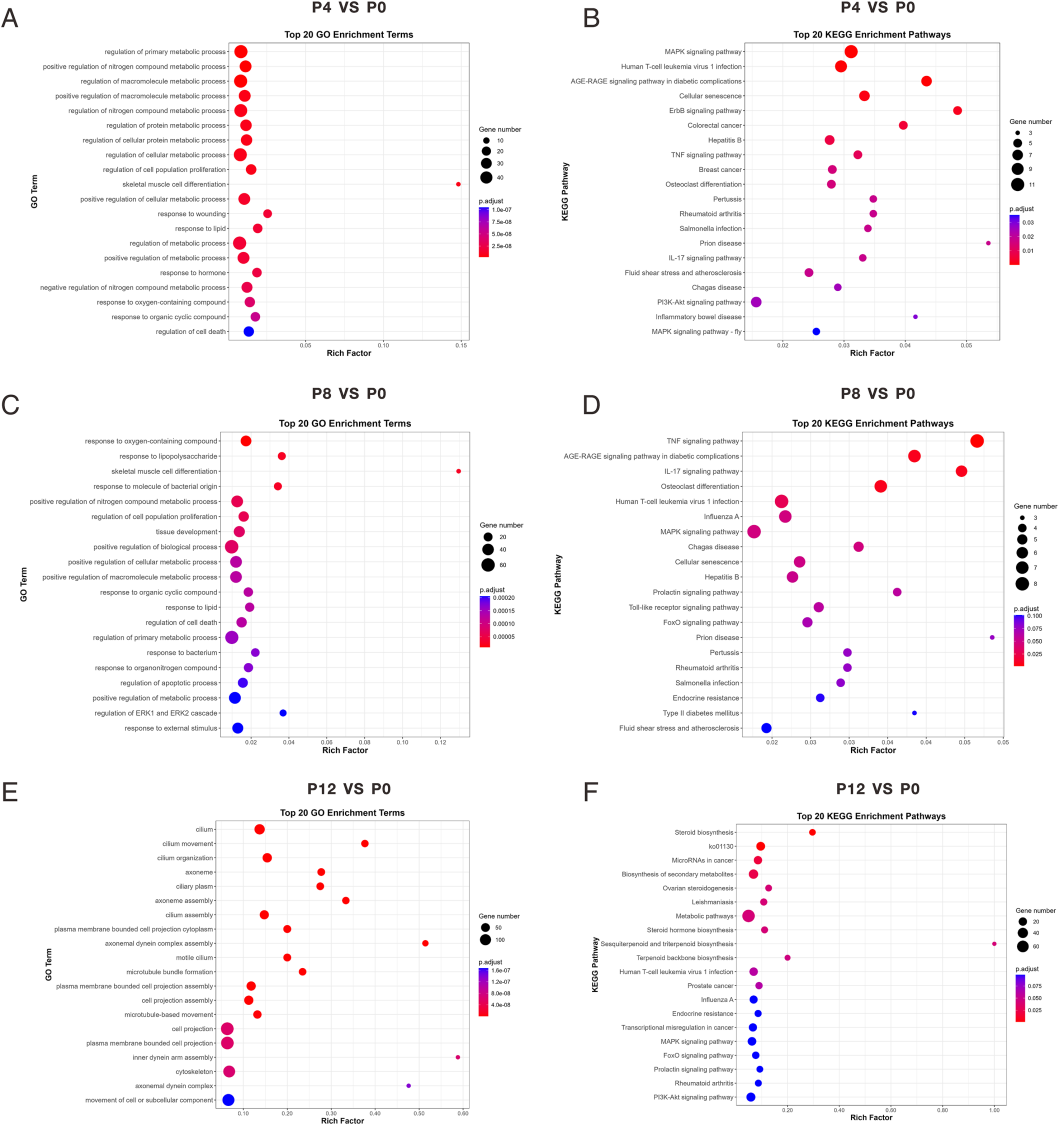
GO and KEGG enrichment analyses of DEGs in PK15 cells at different time points post PRV infection. **(A, C, E)** Top 20 Gene Ontology (GO) enrichment terms of DEGs at 4 h, 8 h, and 12 h post PRV infection compared with uninfected control (P0). **(B, D, F)** Top 20 Kyoto Encyclopedia of Genes and Genomes (KEGG) enrichment pathways of DEGs at 4 h, 8 h, and 12 h post PRV infection.

To further reveal the biological functions of these DEGs, we performed GO and KEGG enrichment analyses. At 4 hpi, GO analysis showed that DEGs were mainly involved in cellular metabolic regulation processes (**Figure 5A**); KEGG analysis indicated enrichment in pathways such as the MAPK signaling pathway, TNF signaling pathway, and IL-17 signaling pathway (**Figure 5B**). At 8 hpi, GO enriched terms included regulation of apoptotic process and positive regulation of cellular metabolic process (**Figure 5C**); KEGG pathways were concentrated in immune and inflammatory response pathways, such as the TNF signaling pathway, IL-17 signaling pathway, and Toll-like receptor signaling pathway (**Figure 5D**). By 12 hpi, GO results showed DEGs were primarily enriched in processes related to cell movement and structure, such as cilium movement and cytoskeleton (**Figure 5E**); KEGG analysis showed significant enrichment in key signaling pathways like the MAPK signaling pathway and PI3K-Akt signaling pathway (**Figure 5F**). These findings indicate that as the infection advances, the host cell’s transcriptional response transitions from initial metabolic stress to intermediate immunological regulation, culminating in intricate biological processes associated with structural remodeling and signal transmission.

### Integrated analysis of ATAC-seq and RNA-seq

**Figure 6:**
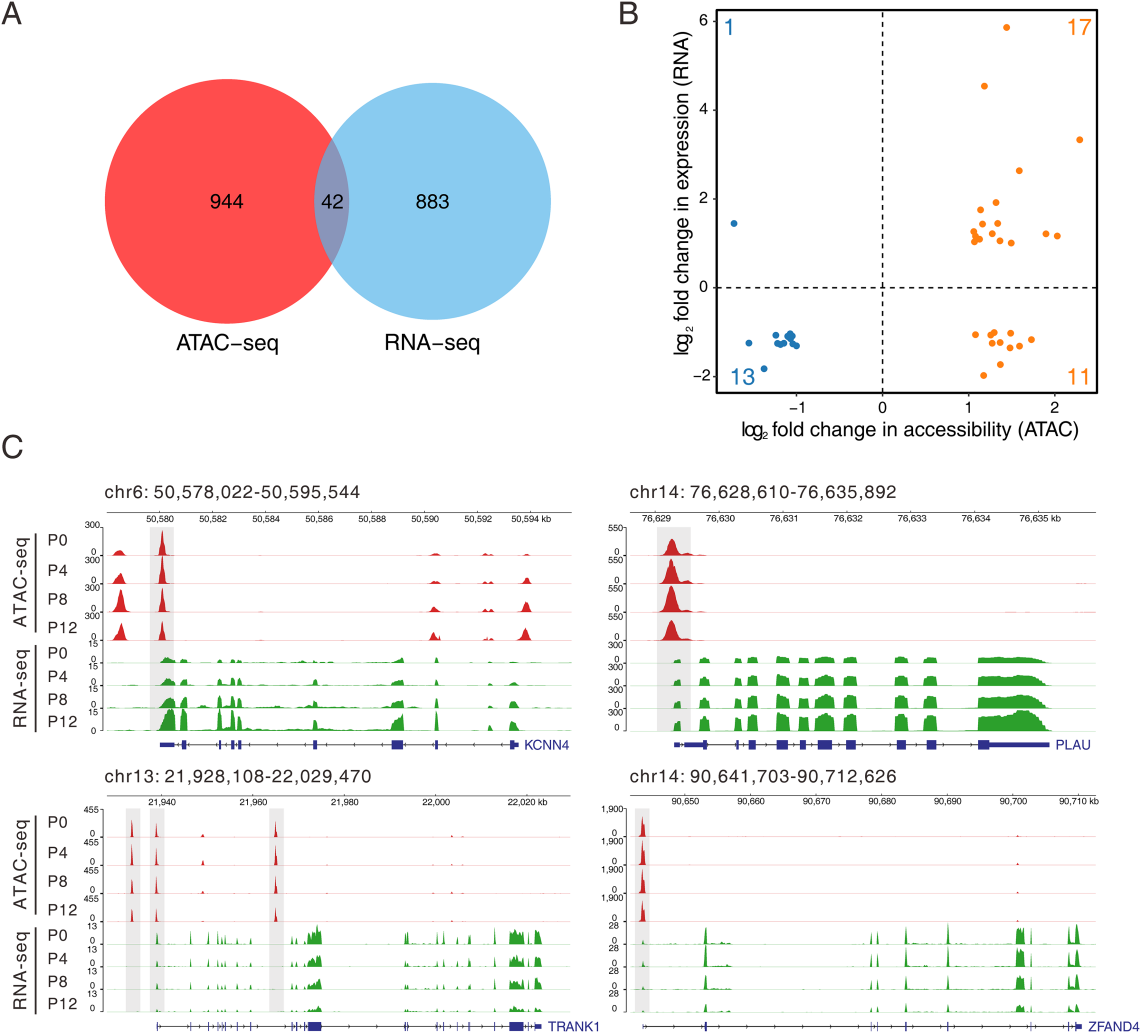
Integrated analysis of ATAC-seq and RNA-seq data. **(A)** Venn diagram showing the overlapping genes identified in ATAC-seq and RNA-seq datasets between PRV-infected (P12) and uninfected (P0) PK15 cells. **(B)** Scatter plot displaying the correlation between changes in chromatin accessibility (x-axis) and gene expression (y-axis) of the overlapping genes. **(C)** Genome browser views showing representative loci (KCNN4, PLAU, TRANK1, and ZFAND4) with coordinated changes in chromatin accessibility (ATAC-seq, red) and transcriptional activity (RNA-seq, green) at different time points post PRV infection.

To explore the relationship between host chromatin accessibility changes and gene transcription following PRV infection, we performed an integrated analysis of ATAC-seq and RNA-seq data comparing the 12 hpi group with the 0 hpi group. The Venn diagram showed 42 shared genes between those associated with differentially accessible regions and the DEGs (**Figure 6A**). This indicates that for these genes, changes in transcriptional activity post-PRV infection are closely related to chromatin structural remodeling. We further analyzed the correlation between accessibility changes and transcriptional level changes for these 42 common genes. The results showed a positive correlation for 30 genes; that is, increased chromatin accessibility was associated with elevated transcription levels, and decreased accessibility with reduced transcription (**Figure 6B**). Meanwhile, a small subset of genes exhibited an inverse pattern, such as 11 genes that showed increased chromatin accessibility but downregulated expression. To further explore the regulatory mechanism of these genes, we performed motif enrichment analysis on the ATAC-seq peaks associated with these 11 genes. The results indicated that these regions were significantly enriched with bZIP family binding motifs, such as Fos, Fra1, and BATF (**Supplementary Table S1**), rather than binding motifs for classical transcriptional repressors.

We depicted the chromatin and gene expression signals for specific genes (**Figure 6C**). At loci such as KCNN4 and PLAU, both ATAC-seq and RNA-seq signals gradually increased with infection time, indicating that the transcriptional activity of these genes significantly increased alongside progressive chromatin opening. Conversely, at the TRANK1 and ZFAND4 loci, both signals gradually decreased over time. Visualization of chromatin accessibility and transcription levels for inflammation and antiviral-related genes showed that at inflammatory factor loci like IL6, IL1A, and CXCL8, ATAC-seq signals significantly increased with infection time, accompanied by a synchronous rise in RNA-seq signals. This suggests that virus-induced chromatin opening promotes the transcriptional activation of inflammatory genes. The immune-regulatory gene SOCS3 and the transcription factor FOSL1 exhibited analogous patterns, with enhanced accessibility correlating with heightened transcription levels. Furthermore, the virus infection associated ssc-miR-221 exhibited enhanced accessibility and transcriptional signals following infection (**Supplementary Figure 4**). These findings illustrate a close interplay between host chromatin structural remodeling and gene transcriptional activation triggered by PRV infection.

### RT-qPCR validation of RNA-seq data

**Figure 7:**
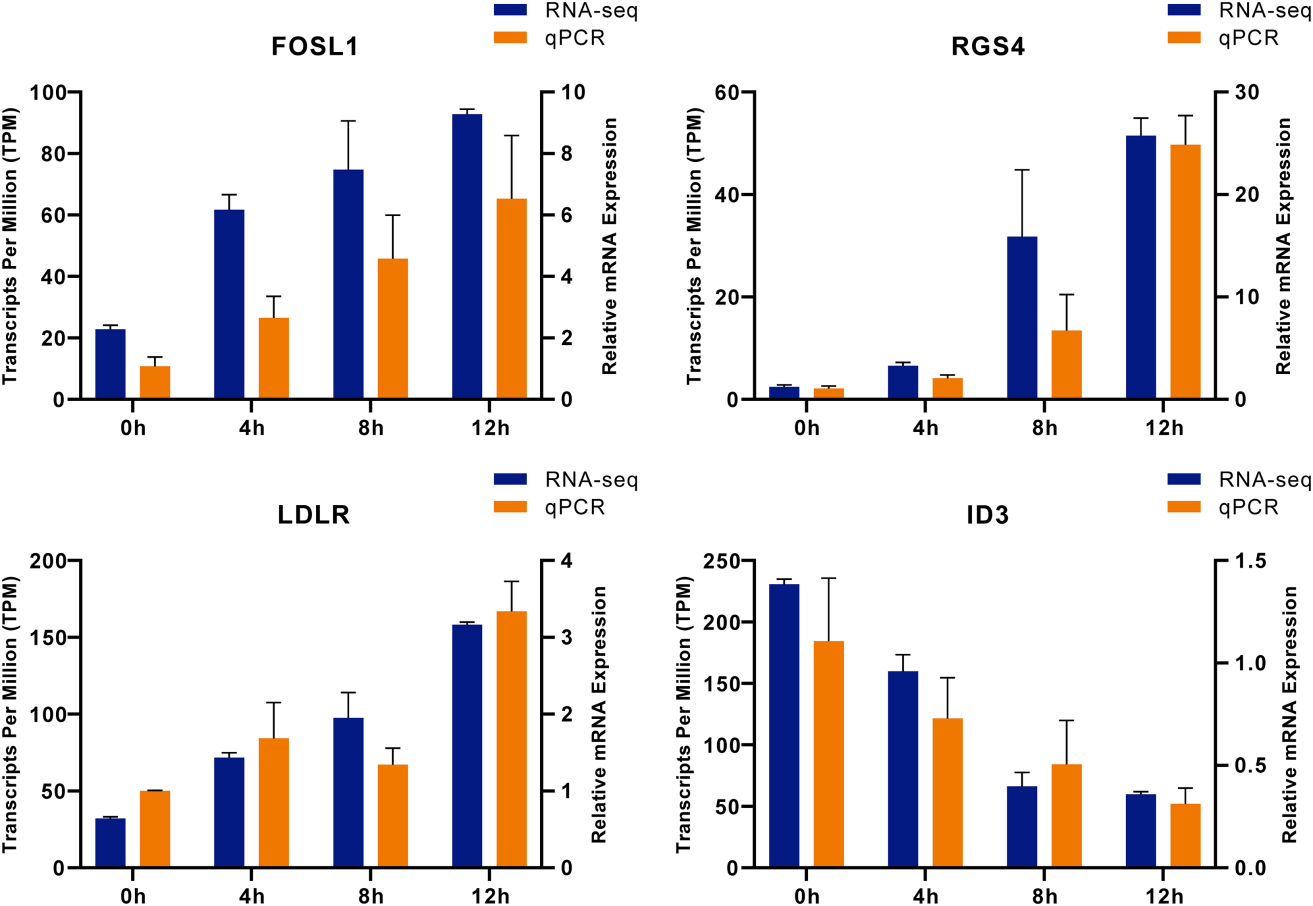
Validation of RNA-seq data using RT-qPCR. Expression profiles of four genes FOSL1, RGS4, LDLR, and ID3 are shown. The x-axis represents different time points (0 h, 4 h, 8 h, 12 h). The left y-axis (blue bars) represents the transcripts per million (TPM) from the RNA-seq data, while the right y-axis (orange bars) indicates the relative mRNA expression levels determined by RT-qPCR. Data are presented as mean ± SEM (n=3).

To verify the reliability of the RNA-seq data, we randomly selected 4 DEGs identified in the transcriptomic analysis, including FOSL1, RGS4, LDLR, and ID3, for RT-qPCR validation. The quantitative results from RT-qPCR showed high consistency with the expression trends observed in the RNA-seq data (**Figure 7**), indicating that the RNA-seq data are highly credible.

## Discussion

The dynamic packing and remodeling of host cell chromatin, combined with the consequent changes in chromatin accessibility, comprise a critical mechanism for controlling gene transcription (43). Pathogens have developed various strategies to interfere with or take control of the host’s epigenetic modification systems through a prolonged evolutionary arms race with hosts. This results in chromatin remodeling that either suppresses the host’s immune responses or activates pathways advantageous for the pathogen’s replication (44, 45). Consequently, studying the mechanisms by which viral infection alters host chromatin accessibility and its correlation with gene transcription is crucial for comprehending viral pathogenesis and formulating innovative antiviral therapies. This study employs PRV, a pathogen responsible for considerable economic losses in the swine industry, as a model to examine the dynamic alterations in host chromatin accessibility and their effects on gene transcription during PRV infection, utilizing ATAC-seq and RNA-seq technologies for the first time.

PRV and HSV-1 are both members of the *Alphaherpesvirinae* subfamily. Previous research has demonstrated that HSV-1 infection induces extensive host chromatin condensation by commandeering host RNA polymerase II and topoisomerase I (46). In this study, our immunofluorescence analysis also detected significant chromatin condensation and aggregation in nuclei at 12 and 24 hpi, particularly at 24 hpi, where virtually all cells had chromatin condensation. This suggests that PRV infection may share a similar mechanism of inducing host chromatin condensation with HSV-1. Moreover, at 24hpi, cells displayed pronounced CPE, suggesting that the chromatin configuration at this juncture likely signifies the final step of cellular demise. On the other hand, our earlier study showed that by 12 hpi, viral genome replication had reached significant levels (47), CPE was not yet obvious, and chromatin condensation appeared in only a small fraction of cells. Therefore, to elucidate the dynamic process of virus-induced chromatin remodeling, we selected time points at 4, 8, and 12 hpi, during which cells had not yet demonstrated evident CPE or extensive chromatin aggregation, for sequencing investigation.

ATAC-seq analysis revealed a significant increase in the number of genome-wide peaks following PRV infection, indicating that PRV infection enhances host chromatin accessibility. In terms of quality control, the discovered peaks were mostly found in promoter regions around transcription start sites, which is in line with the usual pattern of open chromatin areas. This shows that the ATAC-seq data is reliable. Further transcription factor motif enrichment analysis revealed that motifs of Basic leucine zipper (bZIP) family members, such as BATF, ATF3, and AP-1, were significantly enriched in regions with markedly increased accessibility. bZIP family transcription factors play a dual role in viral infection. On one side, they are important parts of the host immune response. For example, BATF is necessary for effector CD8^+^ T cells to become distinct during persistent viral infection (48, 49). Conversely, numerous viruses have developed strategies to exploit bZIP factors; for instance, ATF3 inhibits host antiviral signaling pathways to facilitate viral replication during Japanese encephalitis virus (JEV) infection (50). Therefore, the precise regulation mechanisms of bZIP factors in PRV infection necessitate further comprehensive examination.

Footprint analysis further validated the activation of bZIP family transcription factors during PRV infection. The binding activity of factors such as FOSL1, FOSL2, and BATF increased significantly post-infection. Furthermore, this activation exhibited no significant variation among 4, 8, and 12 hpi, suggesting that these transcription factors are active at the initial stage of viral infection and maintain their functionality throughout the process. In contrast, the binding activity of transcription factor HNF1A decreased significantly at 12 hpi. HNF1A acts as a negative regulator during viral infection, suppressing the innate immune response by inhibiting TBK1 activation and the subsequent interferon (IFN) pathway (51). Therefore, we speculate that the observed reduction in HNF1A binding activity at 12 hpi may be a countermeasure employed by the host to resist viral infection and initiate immune responses.

Transcriptome sequencing results revealed the dynamic transcriptional response of the host to PRV infection. As the infection advanced, the quantity of DEGs rose markedly, displaying unique temporal patterns. In the initial stages (4 and 8 h), differentially expressed genes were mostly characterized by upregulated genes; however, by 12 h, this trend transitioned to a predominance of downregulated genes. This indicates that in the intermediate to later phases of infection, host cell transcriptional programs start to experience extensive repression, potentially aligning with the initiation of chromatin condensation noted at 12 hpi. Functional enrichment analysis indicated that DEGs were predominantly associated with metabolic and inflammatory response pathways. Our previous proteomics study also indicated the presence of numerous metabolic-related differentially expressed proteins following PRV infection (22). Studies have shown that viruses universally reprogram host cell metabolic profiles to create favorable conditions for their own replication. Various viruses can activate essential host pathways, including glucose metabolism, lipid synthesis, and amino acid metabolism, to augment energy supply, raw material synthesis, and viral assembly, so facilitating the creation and release of viral particles (52–54). Moreover, PRV infection elicited alterations in the expression of genes associated with inflammatory responses, including TNF and IL-17, demonstrating that PRV may effectively regulate host inflammation. A study in a mouse model found that PRV modulates the inflammatory response to protect intracranially infected mice from death (55).

A combined investigation of ATAC-seq and RNA-seq validated the synergistic regulatory interplay between chromatin accessibility and gene transcription. The majority of shared differential genes had a positive connection between chromatin accessibility and mRNA expression levels, indicating that enhanced chromatin openness facilitated gene upregulation, and conversely. This corresponds with the traditional paradigm of transcription factor-mediated transcriptional control (9). We also discovered that the correlation between chromatin accessibility and gene expression is not absolutely linear. For instance, we detected inverse regulation in some genes, where 11 genes showed increased chromatin accessibility but downregulated expression. However, we found no enrichment of known transcriptional repressors in the chromatin regions with increased accessibility associated with these downregulated genes. This suggests that the recruitment of specific transcriptional repressors to newly opened chromatin regions, particularly in promoter areas, may not be the primary cause of the widespread transcriptional suppression observed in the late stage of PRV infection. Moreover, at 12 hpi, the upregulated regions in the chromatin accessibility areas were more abundant than the downregulated regions. However, the majority of DEGs were found to be downregulated. This implies the presence of intricate distal regulatory or post-transcriptional regulatory mechanisms. Studies indicate that PRV encodes the conserved alphaherpesvirus virion host shutoff (vhs) protein (UL41), an endoribonuclease that triggers widespread host mRNA degradation, leading to global host shutoff (56). Additionally, there is a time delay between alterations in chromatin accessibility and the increase of mRNA. Transcription always happens after changes in the structure of chromatin, and transcripts need to be processed (57). Therefore, mRNA levels detected at a specific time point effectively reflect the chromatin state of an earlier time point, which may result in a degree of lag when analyzing the association between mRNA levels and chromatin accessibility changes. For instance, although the chromatin accessibility signal of PLAU decreased at 12 h, its mRNA levels remained at a peak for a short period due to the relative time lag of transcription. Despite this lag, the integrated analysis in this study overall reveals consistency between PRV-induced chromatin accessibility remodeling and host transcriptional levels. While this study implicates bZIP family members as key regulators through motif enrichment and footprinting analysis, functional validation remains to be established. Future studies should employ siRNA-mediated knockdown or CRISPR/Cas9 knockout to target candidate factors like AP-1, which will be essential to determine whether their roles in PRV infection are proviral or antiviral. Additionally, chromatin immunoprecipitation sequencing (ChIP-seq) of these transcription factors during infection would provide direct evidence of their genome-wide binding patterns and validate our motif enrichment predictions. Such mechanistic studies will be crucial for identifying potential therapeutic targets for PRV control.

## Conclusion

This study analyzes the dynamic changes in host chromatin accessibility and gene transcription during PRV infection. PRV infection induces widespread chromatin opening early in the infection and continues to influence chromatin structure throughout the infection process. Footprint analysis revealed significant activation of key transcription factors, such as the bZIP family, indicating their important role in the viral infection. RNA-seq analysis showed that PRV infection significantly impacts the expression of genes involved in metabolism and inflammation. The integrated analysis of ATAC-seq and RNA-seq data revealed a positive correlation between chromatin accessibility and gene expression. This study provides insights into the epigenetic mechanisms underlying the interaction between alpha herpesviruses and their hosts.

## Data Availability

The raw data of ATAC-seq and RNA-seq generated in this study have been deposited in the NCBI Sequence Read Archive database under BioProject accession number PRJNA1369774.
