# Supplementary material for: Dynamic chromatin accessibility and transcriptional landscapes of porcine kidney cells during pseudorabies virus infection
Source: Front Immunol. 2026 Feb 4;17:1773053. doi: 10.3389/fimmu.2026.1773053 (PMC12914951; doi:10.3389/fimmu.2026.1773053)
Supplement: Supplementary file 1 [file DataSheet1.docx]

[Supplementary Information](https://static-content.springer.com/esm/art%3A10.1038%2Fs42003-024-06389-x/MediaObjects/42003_2024_6389_MOESM2_ESM.docx)

**Dynamic chromatin accessibility and transcriptional landscapes of porcine kidney cells during pseudorabies virus infection**

Songbai Yang^#^, Mingyang Dong^#^, Haixin Shi, Xiangchen Li, Han Wang, Xiaolong Zhou^*^, Ayong Zhao^*^

Key Laboratory of Applied Technology on Green-Eco-Healthy Animal Husbandry of Zhejiang Province, College of Animal Science and Technology, College of Veterinary Medicine, Zhejiang A&F University, Hangzhou, China.

^#^These authors have contributed equally to this work

^*^Correspondence authors: zhouxiaolong@zafu.edu.cn, zay503@zafu.edu.cn


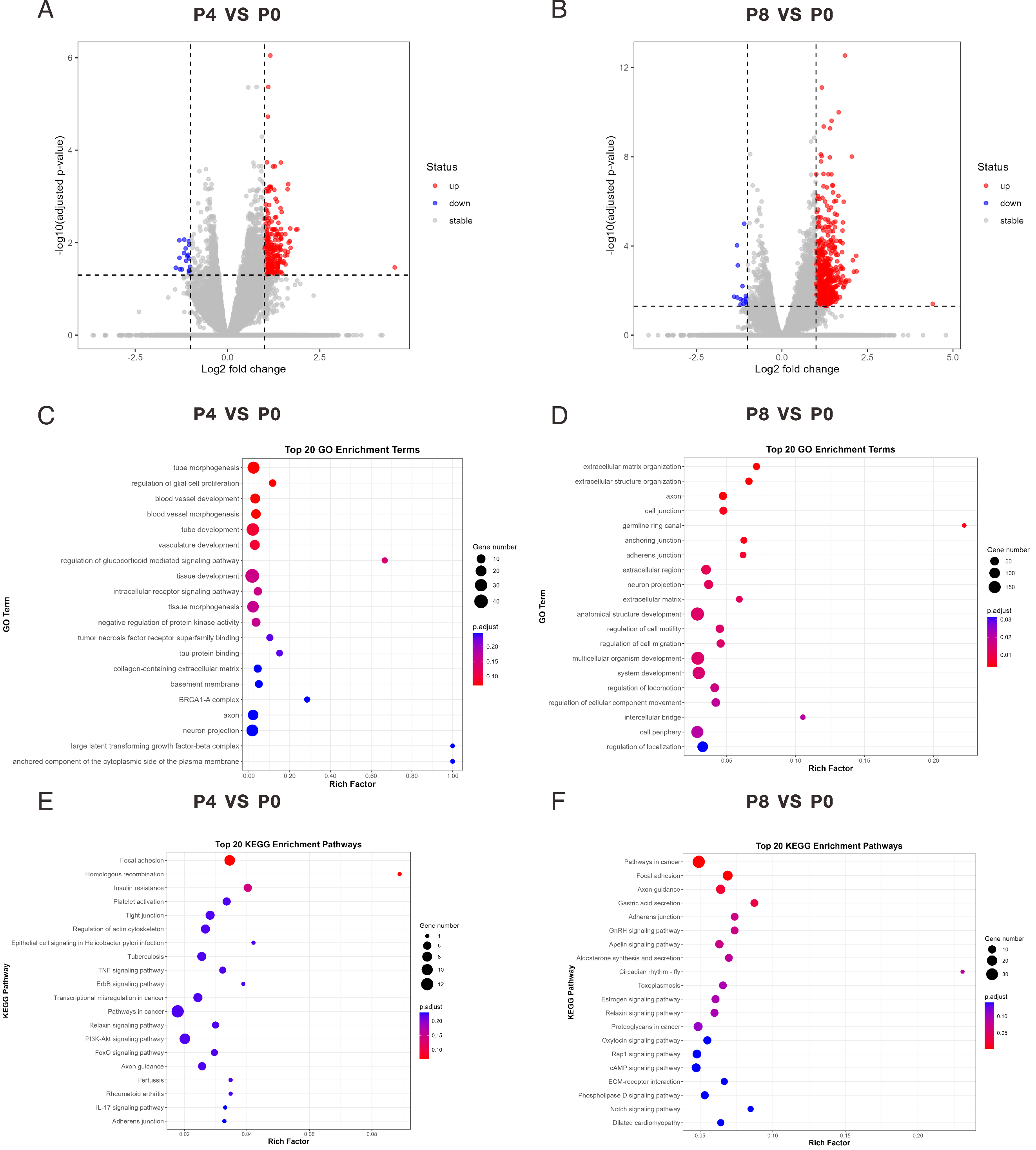


**Supplementary Figure 1. Chromatin accessibility and functional enrichment during PRV infection.**
**(A), (B)** Volcano plots showing differentially accessible chromatin regions (DARs) between PRV-infected (P4 or P8) and uninfected (P0) PK15 cells. Red and blue dots represent up- and down-regulated accessibility, respectively. **(C), (D)** Top 20 enriched GO terms for DAR-associated genes at 4 h (C) and 8 h (D) post-infection. **(E), (F)** Top 20 KEGG pathways enriched for DAR-associated genes at 4 h (E) and 8 h (F) post-infection.


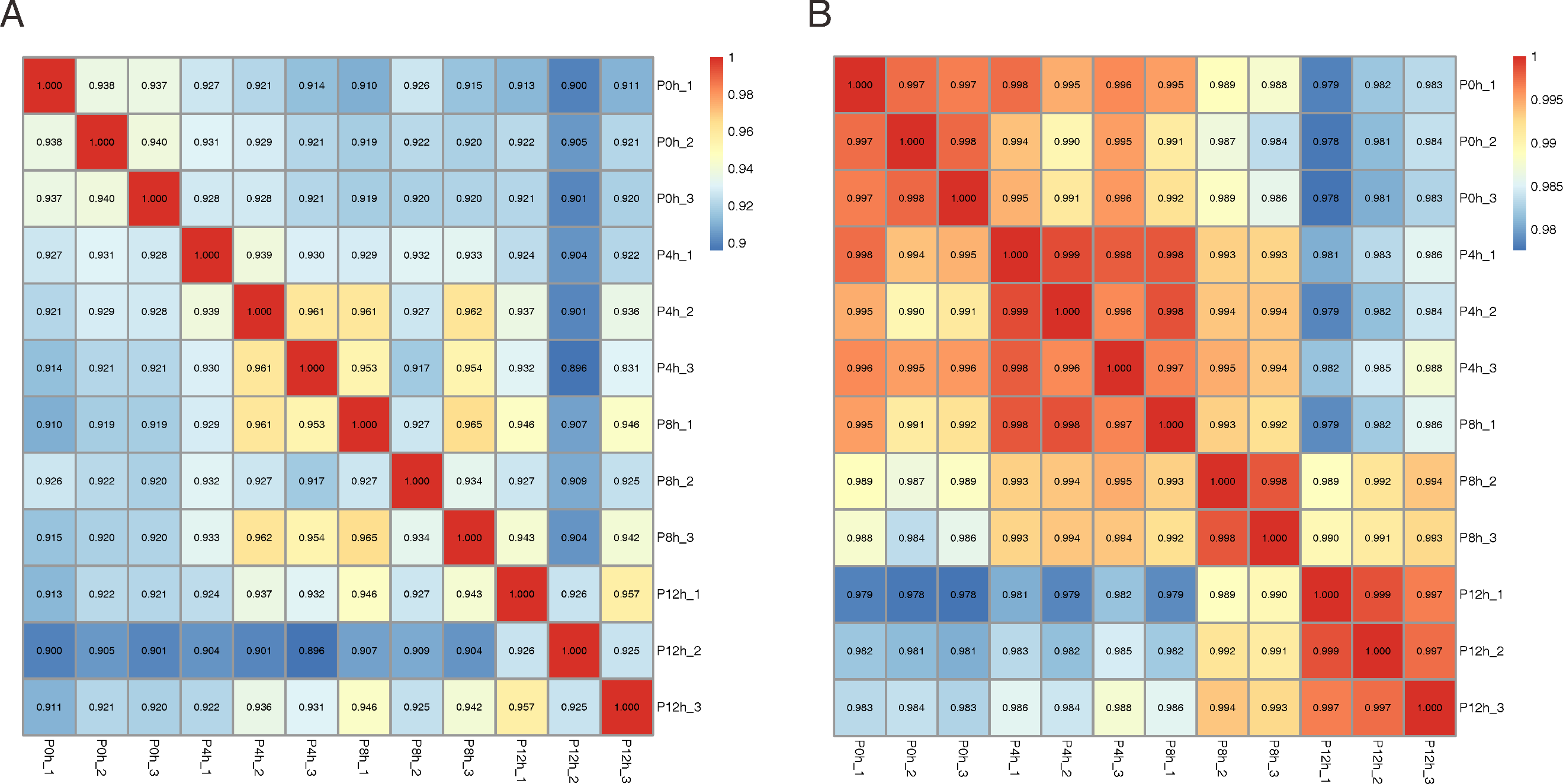


**Supplementary Figure 2. Sample correlation analysis of ATAC-seq and RNA-seq data.**

**(A)** Pearson correlation heatmap of ATAC-seq samples. The correlation coefficients were calculated based on the normalized quantification of filtered peaks. **(B)** Pearson correlation heatmap of RNA-seq samples. The correlation coefficients were calculated based on the gene expression (TPM) matrix.


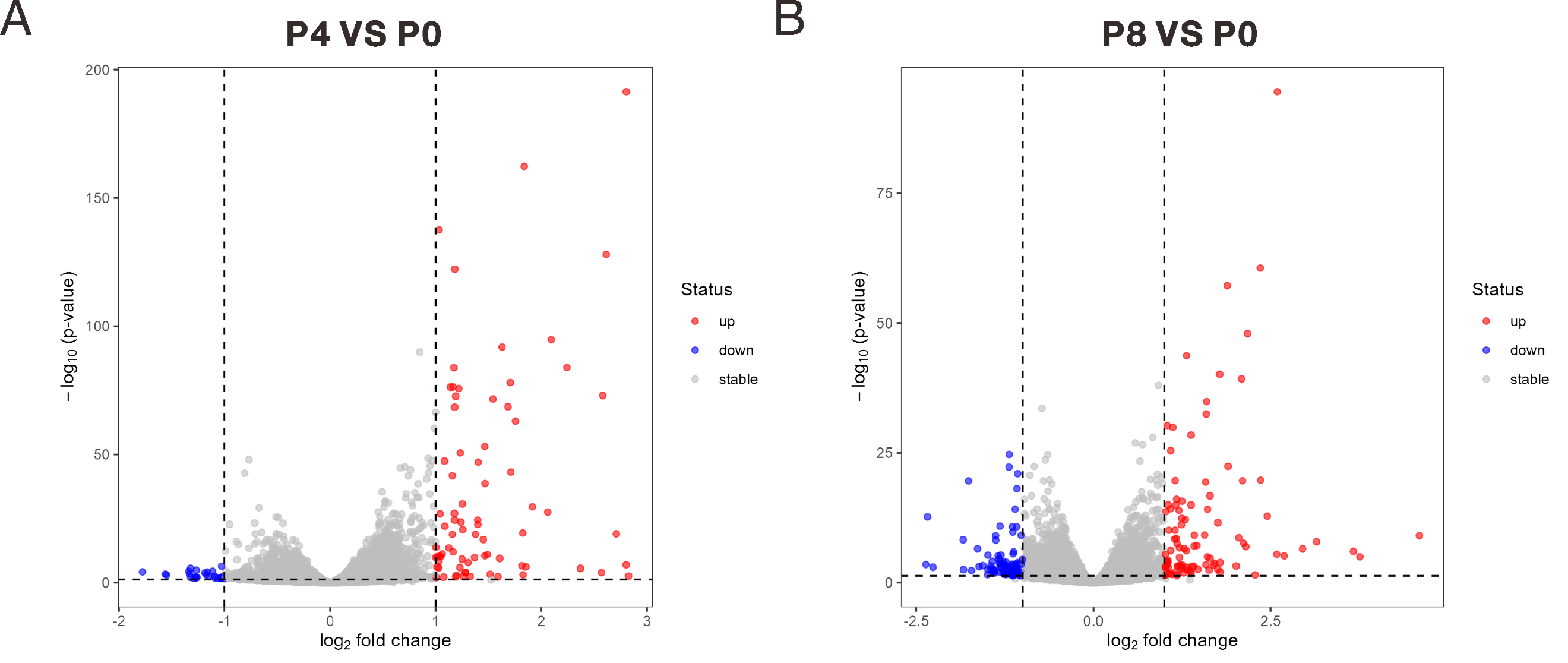


**Supplementary Figure 3. Differential gene expression during PRV infection.**
**(A), (B)** Volcano plots showing differentially expressed genes (DEGs) between PRV-infected (P4 or P8) and uninfected (P0) PK15 cells. Red and blue dots represent up- and down-regulated genes, respectively.


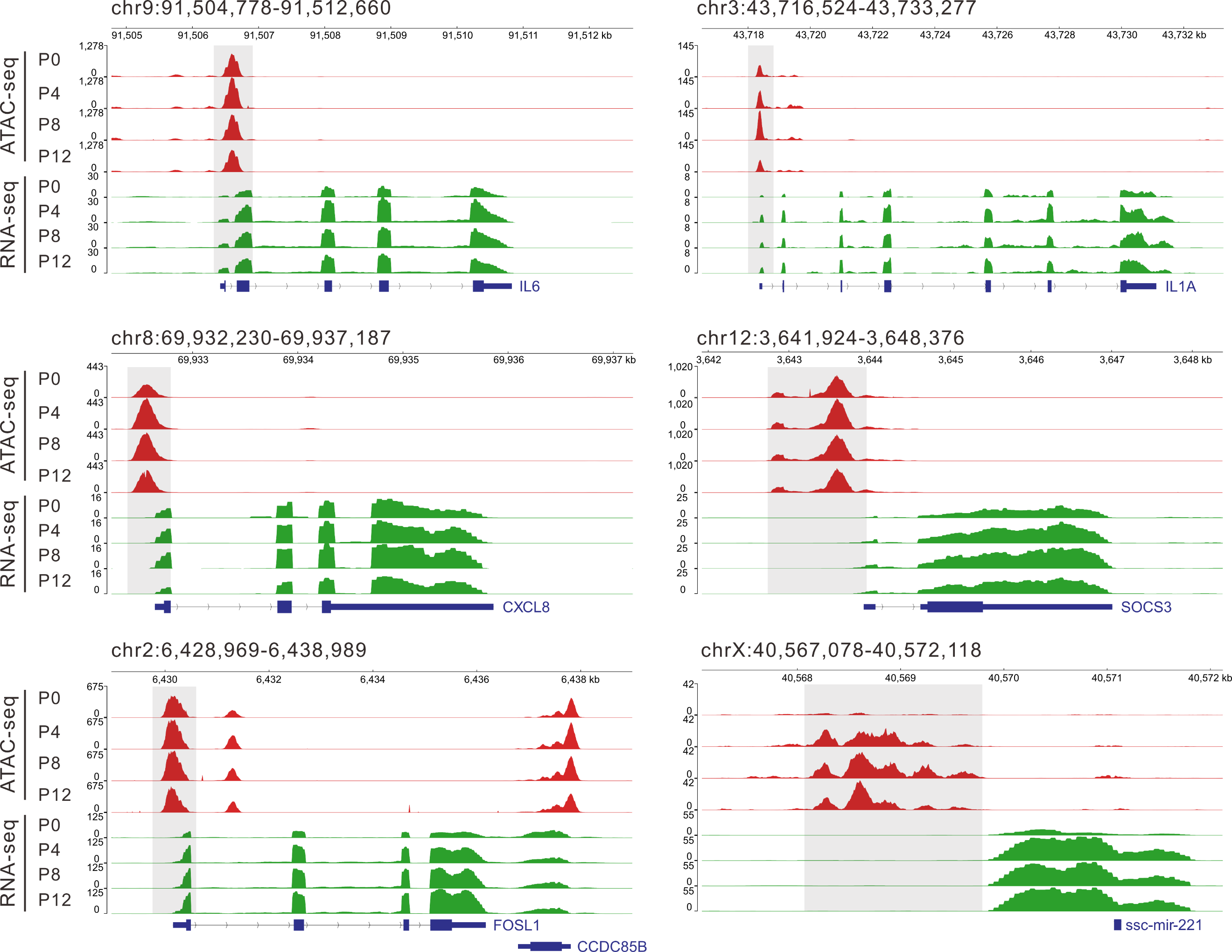


**Supplementary Figure 4. Integrated ATAC-seq and RNA-seq profiles of representative genes during PRV infection.**
Genome browser views showing chromatin accessibility (ATAC-seq, red) and transcriptional activity (RNA-seq, green) at different time points post PRV infection (P0, P4, P8, and P12) in PK15 cells. Regions with increased accessibility and expression are highlighted in gray. Shown are representative loci of IL6, IL1A, CXCL8, SOCS3, FOSL1, and ssc-miR-221, which are associated with inflammatory and antiviral responses.

**Table S1**. Motif enrichment analysis of upregulated ATAC-seq peaks associated with downregulated genes at 12 hpi.

| Name | P-value |
| --- | --- |
| Fos(bZIP) | 1.00E-07 |
| Fra1(bZIP) | 1.00E-05 |
| BATF(bZIP) | 1.00E-05 |
| Atf3(bZIP) | 1.00E-05 |
| Fosl2(bZIP) | 1.00E-03 |
| JunB(bZIP) | 1.00E-03 |
| AP-1(bZIP) | 1.00E-03 |
| Fra2(bZIP) | 1.00E-02 |
